# Retraction: ‘Long non-coding RNA LINC00467 regulates hepatocellular carcinoma progression by modulating miR-9-5p/PPARA expression’ (2019), by Cai *et al.*

**DOI:** 10.1098/rsob.230405

**Published:** 2023-11-03

**Authors:** Open Biology editorial team

**Keywords:** LINC00467, miR-9-5p, microRNA sponge, PPARA, hepatocellular carcinoma

*Open Biol.* **9**, 190074 (Published online 4 September 2019). (https://doi.org/10.1098/rsob.190074)

Following an investigation, we have found that the published manuscript has significant overlap with published manuscript [1] in the text and figures (notably figure 5*d*. Western blot analysis of PPARA expression following the transfection of synthetic miRNA precursor or NC’). This paper has been identified among of a set of papers that present significant text and data duplication and therefore the data reported in this article are unreliable. The authors have not been able to supply the raw data as requested, thus, the editors consider the conclusions of this article to be invalid. The authors have not responded to correspondence about this retraction.

Prof. Jon Pines FRS

Editor-in-Chief, *Open Biology*
